# Allele-Specific Hormone Dynamics in Highly Transgressive F2 Biomass Segregants in Sugarcane (*Saccharum* spp.)

**DOI:** 10.3390/plants13162247

**Published:** 2024-08-13

**Authors:** Noor-ul Ain, Habiba, Ray Ming

**Affiliations:** 1 Center for Genomics, Fujian Provincial Key Laboratory of Haixia Applied Plant Systems Biology, Fujian Agriculture and Forestry University, Fuzhou 350002, China; nooruainali001@gmail.com; 2 Department of Biological Sciences, Lehman College, City University of New York, 250 Bedford Park Boulevard West, Bronx, NY 10468, USA; fnu.habiba79@login.cuny.edu

**Keywords:** sugarcane biomass, extreme segregants, gene duplications, auxin, jasmonic acid, abscisic acid

## Abstract

Sugarcane holds global promise as a biofuel feedstock, necessitating a deep understanding of factors that influence biomass yield. This study unravels the intricate dynamics of plant hormones that govern growth and development in sugarcane. Transcriptome analysis of F2 introgression hybrids, derived from the cross of *Saccharum officinarum* “LA Purple” and wild *Saccharum robustum* “MOL5829”, was conducted, utilizing the recently sequenced allele-specific genome of “LA Purple” as a reference. A total of 8059 differentially expressed genes were categorized into gene models (21.5%), alleles (68%), paralogs (10%), and tandemly duplicated genes (0.14%). KEGG analysis highlighted enrichment in auxin (IAA), jasmonic acid (JA), and abscisic acid (ABA) pathways, revealing regulatory roles of hormone repressor gene families (*Aux/IAA*, *PP2C*, and *JAZ*). Signaling pathways indicated that downregulation of *AUX/IAA* and *PP2C* and upregulation of JAZ repressor genes in high biomass segregants act as key players in influencing downstream growth regulatory genes. Endogenous hormone levels revealed higher concentrations of IAA and ABA in high biomass, which contrasted with lower levels of JA. Weighted co-expression network analysis demonstrated strong connectivity between hormone-related key genes and cell wall structural genes in high biomass genotypes. Expression analysis confirmed the upregulation of genes involved in the synthesis of structural carbohydrates and the downregulation of inflorescence and senescence-related genes in high biomass, which suggested an extended vegetative growth phase. The study underscores the importance of cumulative gene expression, including gene models, dominant alleles, paralogs, and tandemly duplicated genes and activators and repressors of disparate hormone (IAA, JA, and ABA) signaling pathways are the points of hormone crosstalk in contrasting biomass F2 segregants and could be applied for engineering high biomass acquiring varieties.

## 1. Introduction

With the advent of the green revolution and population explosion, the energy demand has risen exponentially. Conventional methods for energy generation, for instance, burning of natural fossil fuels such as gas, coal, and petroleum, are diminishing. Moreover, they have a higher carbon footprint and add their share of greenhouse gases (GHGs), leading to warming and climate change [1]. Thus, migrations toward environment-friendly and sustainable energy systems have become inevitable and a promising area of research. Biofuels contribute (9.4%) as a fourth major source of energy, followed by oil (30.9%), coal (26.8%), and natural gas (23.2%), and is gaining special attention as a critical renewable energy source in local areas worldwide [2]. One of the important feed stocks of biofuels is the biomass generated by plants as carbohydrate “building blocks” at the cost of ambient air CO_2_ and soil water [3]. Panicoid grasses, being C4 plants, are most popular as feedstocks, and their starch and lignocellulosic polymers are converted into monosaccharides prior to fermentation for bioethanol production. Sugarcane is an efficient accumulator of biomass and is considered a benchmark in first and second-generation biofuels [4]. *Saccharum officinarum* (*S. officinarum*), being a member of the *Saccharum genus*, is cultivated and highly efficient in producing and storing sugars. Among the two wild species, *S. robustum* and *S. spontaneum*, the former was once believed to be the ancestor of *S. officinarum*, but recent genomic analyses of these three species proved otherwise [5]. These two wild species, *S. spontaneum* and *S. robustum*, have been extensively involved in breeding programs for different agronomic traits [6]. Cultivated sugarcane has many production constraints such as extended vegetative growth period, high input demands, and lower sugar recovery and growth responses due to different biotic and abiotic stresses. The genome complexity due to the polyploid nature of sugarcane is a primary reason for hindrance in variety development via conventional breeding. Modern sugarcane breeding methods can help develop a framework to dissect biological mechanisms for the improvement of important traits. As it is a principal sugar-producing crop, major research challenges were stagnant yield, lower sugar recovery, and disease resistance [7]. Moreover, extensive research was focused on sugar-related traits in sugarcane, but there are knowledge gaps in developing high biomass sugarcanes, which are referred to as “energy canes” [8].

Phenotypically, biomass increase in sugarcane is achieved by increasing stalk number (SN), stalk height and weight (SH, SW), and stalk diameter (SD). During the previous three decades, advancements in molecular and genetic studies have uncovered a plethora of genes and transcription factors acting in growth and development [9]. Biomass increase is a complex trait; regulation of different pathways in plants contributes to substantial hormonic growth yielding increased biomass, e.g., cell cycle, intrinsic hormone regulation, transcription factors, cell wall synthesis, circadian rhythms, and certain other genetic controls, which contribute to accelerated growth. A number of genetic regulatory mechanisms act to control these traits; sugarcane, being the complex polyploid, involves the interplay of additive and non-additive effects and the interaction of different alleles. Besides molecular cloning, a number of population-based studies have been executed to unravel quantitative trait loci (QTLs) and SNPs controlling biomass yield components such as SH, SN, SD, and brix from interspecific hybrid populations [10].

Plant growth and development are influenced by hormones, particularly auxin, which acts in coordination with other hormones depending on the cell type in response to both developmental and environmental cues. Auxin (IAA) is an important hormone that exerts pleiotropic effects on growth-related processes in spatio-temporal patterns of regulation [11]. Several gene families regulate the biosynthesis, transport, and signaling of auxin in plants. For example, *YUC*, *PIN*, *LAX*, *Aux/IAA*, *GH3*, *ARF*, *SAUR*, and *LBD*. *Aux/IAA* genes, being negative regulators, contribute to the diversity of auxin-induced responses in different organs and at various developmental stages. *Aux/IAA* proteins repress auxin-mediated gene regulation by binding Auxin Response factors (ARFs) through their conserved domains. SAUR genes are early auxin-responsive and encode highly unstable mRNAs. Gretchen Hagen3 (*GH3*) proteins play a role in the conjugation of amino acids to various acyl substrates and thus maintain the homeostasis of jasmonic acid (JA) and IAA [12]. *SAUR*s play roles in cell elongation and increase in biomass [13]. Mutations in different *AUX-IAA* genes such as *IAA14/SLR* [14], *IAA17*/*AXR3* [15], *IAA19/MSG2* [16], and *IAA28* [17] in *Arabidopsis* showed altered responses to auxins and modified plant morphology.

Similarly, jasmonic acid (JA) is a prototypical member of a class of related oxylipins, which are collectively termed as jasmonates and are important signaling molecules in the stress and anti-oxidant response [18]. JAZ-related genes with the *ZIM* domain regarded as *TIFY* genes are the repressors of JA signaling and are degraded by the SCF complex to initiate the transcription of JA-responsive genes. CORONATINE INSENSITIVE 1 (*COI1–JAZ*) complex acts as a substrate for the perception of incoming jasmonic acid (JA–Ile), and after ubiquitination of JAZ repressors (*TIFY*), *MYC* transcription factors initiate the transcription of JA-responsive genes [19]. Regarding abscisic acid, the three primary proteins regulate the signaling and expression from ABA perception to transcription of *ABA*-responsive genes. These three pillars include pyrabactin resistance 1 (*PYR*)/PYR1-like (*PYL*)/regulatory components of ABA receptor (*RCAR*), protein phosphatase 2C (*PP2C*), and sucrose nonfermenting-1 (*SNF1*)-related protein kinase 2 (*SnRK2*) [20]. When cellular ABA level is very low under normal growth conditions, group 2 Protein Phosphatases (*PP2C*) genes dimerize with subclass III *SnRK2*s and inhibit their kinase activity. Under stress conditions, biosynthesis of ABA takes place, and *PYR/PYL/RCAR* receptors cause the inhibition of *PP2C*s, enabling the activation of *SnRK2*s kinases and regulating the downstream genes and TFs [21].

In the study, *S. officinarum* “LA Purple” was crossed with *S. robustum* “MOL5829”, each with a chromosome number of 2n = 8× = 80. They were selected as representative genotypes of the two species. We generated an F2 population from self-fertilized F1 plants, and multiple after rounds of field trials and phenotypic data showed two distinct extreme biomass groups. Therefore, to decipher the genetic controls and understand the growth processes underlying heterosis, we performed an RNA-Seq analysis of F2 introgression segregants of high and low biomass and identified different functional categories for biomass regulation. Using the allele-specific reference genome of *S. officinarum*, we identified that hormone-related gene models (GMs), alleles, paralogs, and tandemly duplicated genes controlled by their native promoters provide regulatory role in biomass accumulation in extreme segregants of this F2 population.

## 2. Results

### 2.1. Sequence Read Alignment and Differential Expression Analysis

RNA-Seq data were generated from 22 extreme segregants of F_2_ population derived from an interspecific cross between *S. officinarum* “LA Purple” and wild species *S. robustum* MOL5829, including 14 high biomass and eight low biomass segregants (Supplementary file 1; Table S1). Extremely low and high biomass hybrids ranged between 18.0 to 37.0 and 78.7 to 106.5 metric tons/hectare of 12-month-old plants, respectively (Figure S1). After mRNA extraction from the third, sixth, and ninth internodes and leaf, cDNA libraries (combined of all plant sections) were constructed and used for RNA sequencing. The details of phenotypic data and sugar contents (reducing and non-reducing) were previously reported, and the sugarcane transcriptome was analyzed using the *Sorghum bicolor* L. Moench as a reference genome due to the unavailability of the high-quality sugarcane reference genome at that time [22]. Although the small genome of sorghum is an attractive model for tropical C4 photosynthetic grasses, it has a diploid genome and minimal duplication events in the genome. The genome of *Saccharum* species is highly polyploid and aneuploid, exhibiting variable ploidy levels among different loci, a large genome size, and abundant repetitive sequences [23]. Comparing sugarcane transcript data with sorghum would compromise the contribution of allelic and duplicated copies of gene models, which are equally contributing to polyploid crops. Therefore, we re-analyzed our Illumina raw sequences of 22 RNA-seq samples (34 GB, 293.5 million reads, and a length of 151 nucleotides) with a high-quality allele-specific *S. officinarum* reference genome. After quality trimming and removing the adapter sequences, data were reduced to 19 GB and 191.3 million clean reads. Although HISAT2 is an efficient aligner with better performance in terms of a high percentage of correctly aligned reads and high-precision rates, in our data, STAR outperformed in alignment with a higher alignment rate (~85%) in all the samples, followed by transcriptome assembly and differential expression analysis.

**Figure 1:**
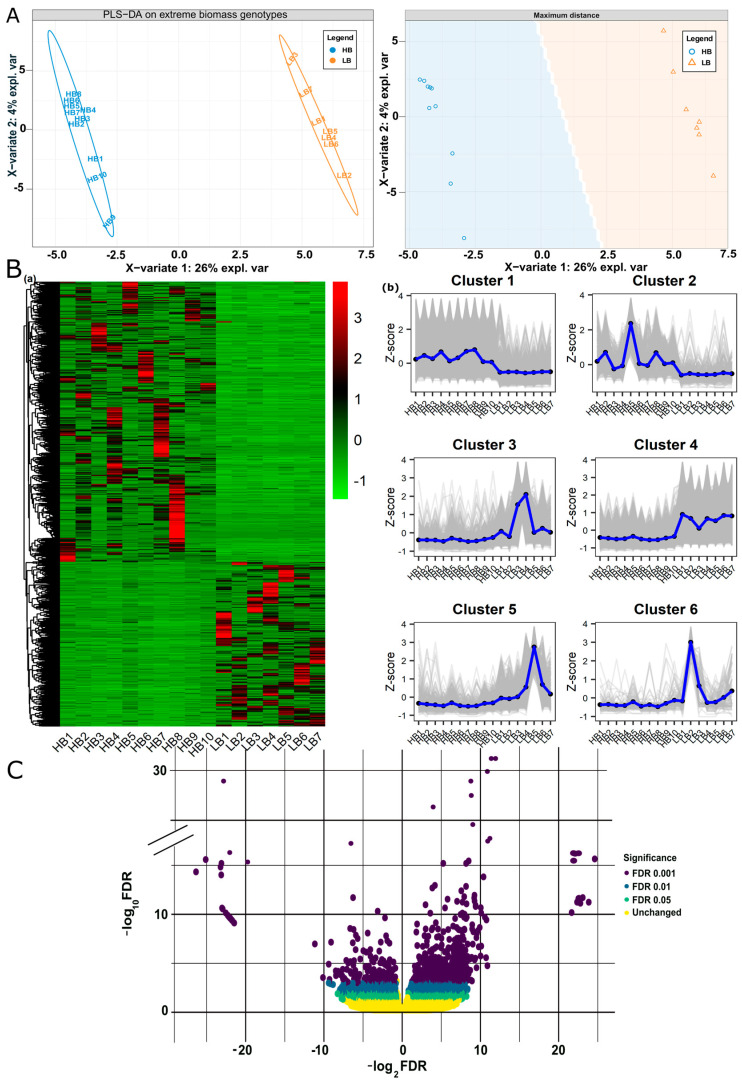
Partial least squares-discriminant analysis (PLS-DA), Hierarchical clustering, and volcano plot of DEGs: (**A**) PLS-DA score plot of FPKM data of DEGs generated by using preprocessed original data shows the clustering of extreme biomass segregants. Component 1 (26%) clearly distinguishes the two biomass groups. (**B**) (**a**) Hierarchical clustering heatmap of the DEGs based on FPKM expression values, (**b**) Six clusters indicating the up and down regulated genes identified in heatmap. (**C**) Volcano plot overall shows the range of log2FC of DEGs, i.e., 24 to −26 from right to left.

Among twenty-two samples, four samples from high biomass and one sample from the low biomass group were excluded, being outliers in the clustering analysis of samples. Finally, DeSeq2-based log2FC estimation of 17 samples (10 HB, 7 LB) resulted in 174,593 genes and alleles (GMs (gene models), alleles, tandem duplicates, and paralogs, (Supplementary file 1; Figure S2 and Table S1). We sorted differentially expressed genes using the thresholds, FDR |≤0.05| and *p*-val |<0.01| while log2FC |>2| for upregulated and log2FC |<−2| for downregulated. Considering the above criteria of selection, 8059 genes and alleles were regarded as DEGs, 5540 were upregulated, and 2519 were downregulated in high biomass samples (Supplementary file 2; Table S3). Among these 8059 genes and alleles, 21.5% belonged to GMs, 68% was an allelic contribution, 0.14% were tandem duplicates, and 10% were paralogs. To visualize expression patterns in samples based on FPKM and log2FC, we employed Partial Least Squares Discriminant Analysis (PLS-DA), hierarchical clustering, and volcano plot analyses of the differentially expressed genes. PLS-DA plot reduced the dimensions of large data and showed distinct differences in gene expression patterns among low and high biomass segregants. PC1 (26%) exhibited maximum variation in the clustering patterns and showed low biomass samples are tightly clustered on the positive *x*-axis (Figure 1A). The Hierarchical clustering heat map showed variability between two biomass groups, and it grouped all the genes in six clusters using expression values (Figure 1B). The volcano plot visualization showed an overall trend of fold change in high biomass segregents, i.e., 24 to −26 (Figure 1C).

### 2.2. Exploration of Biological Processes Involved in Growth and Biomass by GO

The GO enrichment analysis performed on differentially expressed genes (DEGs) in high biomass samples compared to low biomass samples revealed significant enrichment in several key biological processes and cellular components. Notably, the “response to stimulus” (GO:0050896) term, involving 667 genes, showed a *q-value* (0.001) and accounted for 36.32% of the DEGs, indicating that high biomass samples are highly reactive to various environmental and internal stimuli. Additionally, the “response to acid chemical” (GO:0001101) term, comprising 414 genes with a *q-value* of 0.000001 and representing 22.54% of the DEGs, suggests that these samples have mechanisms to cope with acidic conditions, essential for their growth and survival in specific environments. In terms of cellular components, the “cell wall” (GO:0005618) term, including 63 genes with a *q*-value of 0.000001 and 4.93% of the DEGs, highlights that high biomass samples may possess robust cell wall structures, contributing to increased stability and protection.

Furthermore, a significant number of genes were enriched in the biological process category in high biomass samples, including the signaling process (GO:0023052) was enriched by 15.56%, the transmembrane transport category (GO:0055085) included 20.43% genes, and cellular nitrogen metabolism biosynthetic process (GO:0034641) included 10.12% of the DEGs. For cellular components, membrane metabolism (GO:0016020) included 8.76%, cell wall (GO:0005618) included 4.93%, and extracellular region (GO:0005576) included 9.432% of the DEGs. Moreover, the genes involved in electron transport and catalysis (GO:0003824; *q-value* < 0.05) were enriched by 12.345%, transmembrane transporter activity (GO:0022857; *q-value* < 0.05) by 14.56%, transferase activity (GO:0016740; *q-value* < 0.05) by 18.234%, and macromolecule modification (GO:0043412; *q-value* < 0.05) included 11.67% of the DEGs. These findings emphasize the complex interplay between environmental interactions and structural adaptations in high biomass samples, providing insights into the biological mechanisms supporting their growth and development (Supplementary file 1; Figure S3, Supplementary file 2; Table S6).

### 2.3. Hormone Dynamics in Extreme Segregants

**Figure 2:**
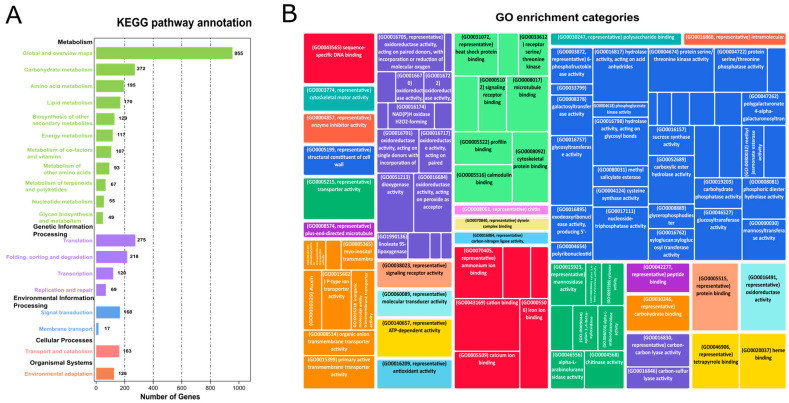
KEGG and GO analysis of DEGs: (**A**) Bar plot for the KEGG categories and enriched DEGs in KEGG analysis. The abscissa depicts enriched pathways while gene number is plotted on an ordinate axis. (**B**) GO shows the enrichment of potential DEGs in different categories, i.e., biological processes, cellular components, and molecular function.

To identify the regulatory role of hormones in growth, cell wall remodeling, and biomass accumulation, KEGG analysis was performed for the functional annotation of DEGs between high and low biomass samples (Figure 2A, Supplementary file 2; Tables S4 and S7). A considerable number of genes were enriched in different metabolic pathways in the KEGG analysis. For example, 96 genes (4.70%) were differentially regulated in signal transduction pathways, 346 genes (16.90%) in carbohydrate metabolism (glycolysis, sugars, starch, pyruvate metabolism), and 42 genes (2.12%) in glycan biosynthesis. Hormone-related genes obtained from the KEGG analysis were categorized into gene models (GMs), allelic copies, and gene duplications (tandem and paralogous duplicates) using an eight-column allele table. Hormone-related genes obtained from KEGG analysis were categorized into GMs (gene models), allelic copies, and gene duplications (tandem and paralogous duplicates) using an eight-column allele table. In polyploid species, alleles are the homologous genes on the homologous chromosomes at the same loci. Tandemly duplicated genes, which originated owing to unequal crossing over, were categorized on the criteria if they had a difference of configurable gene rank = 1. Dispersly duplicated paralogs are neither neighbors nor on the homologous chromosome, and they are re-labeled based on blastp hits [24,25]. The details of all the discussed genes and their respective categories (GMs, alleles, tandem, and paralogous duplicates) are discussed in (Supplementary file 2; Table S5).

### 2.4. Auxin-Related Genes

**Figure 3:**
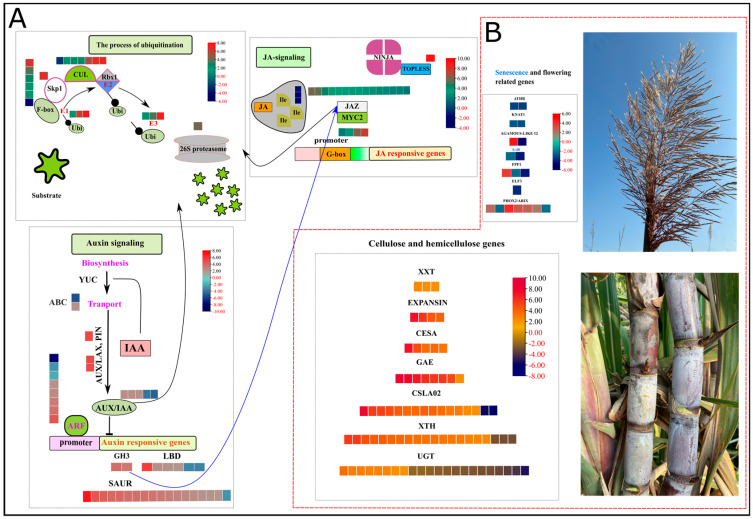
Heatmap of log2FC values in HB segregants. Red and black values in legend show down and upregulated genes. (**A**) shows ubiquitin-mediated signaling of auxin and jasmonic acid signaling pathways. (**B**) depicts the expression patterns of hormone-responsive growth-related genes in cell wall and terminal developmental phases, i.e., inflorescence and senescence. *S-40*: Senescence regulator, *ATHB*: ARABIDOPSIS THALIANA HOMEOBOX 7, *KNAT1*: BREVIPEDICELLUS, *PHOX2/ARIX*: Transcription factor PHOX2/ARIX, *ELF3*: EARLY FLOWERING 3 (*ELF3*), *FPF1*: FLOWERING PROMOTING FACTOR 1, *AGAMOUS-LIKE 12*: AGAMOUS-LIKE 12, *XXT*: galactosyl transferase GMA12/MNN10 family, *EXPANSIN*: EXPANSIN, *CESA*: cellulose synthase subfamily, *GAE*: GDP-mannose 4,6 dehydratase, *CSLA02*: belongs to the glycosyltransferase 2 family, *XTH*: Xyloglucan endohydrolysis (*XEH*) and or endotransglycosylation (*XET*), and *UGT*: UDP-glycosyltransferase family.

Auxin, an essential hormone in modulating growth and development, showed the enrichment of several GMs, alleles, and duplications in high biomass samples. Annotated genes were confirmed using blastp in phytozome and NCBI (https://phytozome.jgi.doe.gov/pz/portal.html (accessed on 21 May 2022)). The sorghum genome, a model for C4 plant species having high evolutionary proximity with sugarcane, has a significant number of auxin-related gene family members, i.e., 26 *Aux/IAA* genes, 16 *GH3* genes, 36 *LBD* genes, and 25 *ARF* genes [26]. Two members (Soffic.03G0032240-1A, Soffic.01G0025830-1P) of auxin influx *AUX 1* (Auxin influx carrier protein) were up and downregulated in HB, whereas one allele (Soffic.05G0006040-2B) related to auxin efflux *PIN* (PIN-FORMED) was upregulated. There was an over representation of five auxin-responsive *Aux/IAA* (repressor) genes (GMs and alleles) in the high biomass group. Two members (Soffic.08G0009450-1P, Soffic.03G0018710-2B) of the GH3 family (indole-3-acetic acid-amido synthetase) showed a profound upregulation in the high biomass group. Eighteen members of the small auxin-up RNAs (SAUR) family and six members encoding lateral organ boundaries (*LBD*) were upregulated in the HB group as compared to the LB group. Among eight differentially regulated *ARF* (AUXIN RESPONSIVE FACTORS) genes, 7 showed upregulation in the HB group. The active contribution of auxin signaling and responsive genes in the HB group predicts auxin-mediated growth changes accounting for high biomass (Figure 3A, Supplementary file 2; Table S5).

### 2.5. Jasmonic Acid (JA) Signaling Pathway Genes

Jasmonic acid is a lipid-derived hormone that regulates important stress and immunity-related responses. Many jasmonic acid-related genes were highly enriched in the signal transduction category identified by KEGG analysis (Figure 3A, Supplementary file 2; Table S5). Coronatine-insensitive (*COI1*) protein acts essentially as a receptor of jasmonoyl–isoleucine (*JA–Ile*), a conjugated form of JA. Three DEGs coding *COI1* were downregulated in the HB group, indicating the absence of substrate for the signaling of jasmonic acid responses. There was an over representation of 15 ZIM-domain (*JAZ*) repressor proteins, among which 13 were upregulated in the HB group. The action of *JAZ*-mediated repression is accomplished by Groucho/Tup1-type co-repressor TOPLESS (*TPL*); one allele encoding TPL is also upregulated in the HB group. Thanks to the upregulation of JA repressor genes, which inhibited the activation of JA downstream, stress-responsive genes inhibit the activation of stress response and allow the normal function of growth-related genes for increased biomass.

### 2.6. Abscisic Acid (ABA)-Related Genes

Regarding the abscisic acid signaling pathway, 23 members were differentially expressed in high and low biomass groups. Type 2C protein phosphatases (*PP2C*s) act as a negative regulator in abscisic acid signaling pathways. Our data showed down and upregulation of six and four *PP2C* coding sequences. One class of protein kinases, i.e., Snf1-related protein kinases 2 (*SnRK2*s), is activated by autophosphorylation after PP2C genes are repressed. The results exhibited that seven *SnRK2* genes showed upregulation while three genes were downregulated. Moreover, two Basic leucine zipper (*bZIP*) and one cis-regulatory DNA element, i.e., abscisic acid (*ABA*) response elements (*ABRE*s) were both downregulated in the HB group, respectively (Supplementary file 1; Figure S4).

### 2.7. Hormone-Responsive Genes Accounting for Growth

Several genes may be induced or suppressed by the exogenous and endogenous hormones in their vicinity; for example, cell wall synthesis CELLULOSE SYNTHASE (*CESA*) and cell wall expansion genes xyloglucan (*XTH*) and Expansins (*EXP*) strongly respond to auxin, brassinolide, and gibberellic acid. Five alleles encoding *CESA* proteins were upregulated in the HB group. Cell wall loosening and expansion are critical for cell growth and secondary cell wall formation. A plethora of *XTH* genes and *EXP* genes were upregulated in the HB group, i.e., *XTH* = 19, *EXP* = 4. Three genes encoding galactosyl transferase, eight genes belonging to GDP-mannose 4, 6 dehydratase, and fourteen members of the glycosyltransferase family were upregulated in the HB group. UDP-glycosyltransferases play their indispensable roles in the glucosylation of aglycones such as hormones, secondary metabolites engaged in stress and defense responses, and xenobiotics. Fourteen genes encoding UGTs were downregulated, whereas 11 genes were upregulated in the HB group (Figure 3B, Supplementary file 2; Table S5).

### 2.8. Flowering and Senescence Genes Downregulated in High-Biomass Group

Flowering is an important developmental transition in plants, and time to flowering induction is a key trait for deciding to assimilate portioning and biomass accumulation. Major integrators of flowering-related pathways are FLOWERING PROMOTING FACTOR 1 (*FPF1*), EARLY FLOWERING 3 (*ELF3*), and AGAMOUS-LIKE MADS-BOX PROTEIN (*AGL12*). Two genes encoding *FPF1*, one *ELF3*, and one *AGL12* were downregulated in the HB group. Expression of certain other genes responsible for inflorescence architecture, for example, HOMEOBOX (WUS)-related genes, BREVIPEDICELLUS, and PROTODERMAL FACTOR 2, was also downregulated in the HB group. Moreover, newly identified gene family members (*S-40*) related to senescence were also downregulated in the HB group, which supplemented the lower activity of flowering-related genes and continued vegetative growth owing to growth-inducing hormones and downstream growth-responsive genes (Figure 3B, Supplementary file 2; Table S5).

### 2.9. Identification of WGCNA Modules Associated with Hormone and Cell Wall Expansion Genes

**Figure 4:**
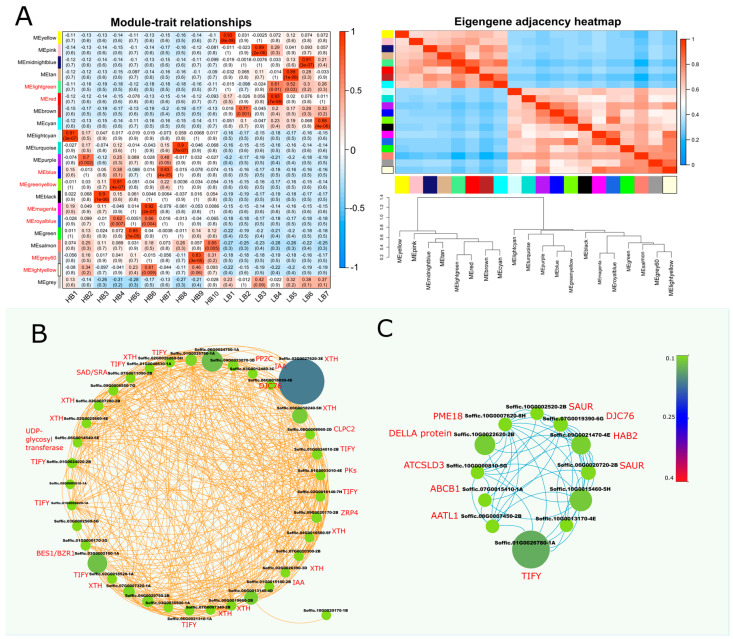
Weighted gene co-expression network analysis (WGCNA) of DEGs between HB and LB segregants. (**A**) eigengene modules, module–trait relationship, and module–module relationship. Module–trait heatmap shows Pearson’s correlation of all the modules with samples, whereas in the module–module relationship, progressive saturation in blue and red color points to high co-expression interconnectedness. Additionally, it shows dendrogram of module clustering, with green and red horizontal lines representing threshold (0.25, 0.3). (**B**) Cytoscape network shows co-expression network of blue module, which depicts highly upregulated genes in HB segregants (**C**) Cystoscape network of overrepresented DEGs in module “greenyellow”. Size and color of nodes are proportional to weights, whereas edge colors correspond to module names.

Weighted gene co-expression network analysis (WGCNA) was performed using 8540 non-redundant genes to identify modules with similar expression patterns. This analysis identified 21 distinct modules, as shown in Figure 4A and Supplementary file 1 (Figure S5). From the module–trait relationship co-relation heat map, “blue” and “greenyellow” modules were selected as highly correlated (r = 0.83, r = 0.91) modules in the HB group. A Cytoscape representation of the blue module genes, with an edge weight over 0.10, indicated a high level of connections between the genes. Blue module network (left) showed the connections between different hormone repressor genes (*IAA*, *TIFY*, and *PP2C*) and cell wall-related growth genes i.e., *XTH* (xyloglucan endohydrolysis (*XEH*) and or endotransglycosylation (*XET*)), UDP-glycosyl-transferase, Histone-lysine N-methyltransferase and O-methyltransferase (Figure 3). The green yellow module network (right) comprised co-expression of *DELLA*, *TIFY*, *SAUR*, *PP2C* (*HAB2*), *ABCB1*, and cell wall-related gene Pectinesterase (*PME18*). The “blue” module, consisting of 1158 genes, and the “greenyellow” module, consisting of 412 genes, appeared to be associated with hormone regulation and biomass gain (Figure 4B,C). These modules represented highly significant gene connections having the merge cut height (~0.25).

**Figure 5:**
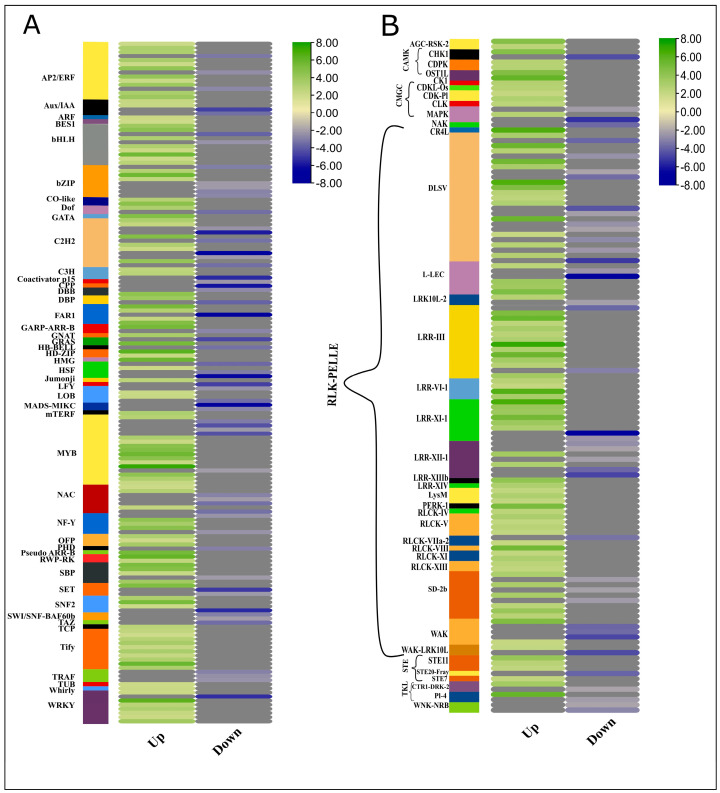
Heatmap generated using log2FC. Expression dynamics of TFs and PKs involved in high biomass samples. Green and blue scales represent up and downregulated TFs in HB samples, whereas grey color indicates blanks.

To apprehend the regulatory roles of transcription factors (TFs) and protein kinases (PKs), we identified TFs from the protein sequences of differentially expressed genes. In plants, DNA transcription involves more than 1500 TFs to regulate target genes by binding with cis-regulatory elements in the promoter region [27]. Mapping the important transcription factors can provide insights into understanding the transcriptional regulation of the plant hormone genes in achieving enhanced biomass (Figure 5A, Supplementary file 2; Table S8). In this RNA-Seq data, a high proportion of TFs of 46 different families were differentially expressed between high and low biomass groups; for example, 45 *WRKY*, 16 *TIFY*, 38 *NAC*, and 18 *GRAS* family TFs were differentially regulated between high and low biomass groups. The over-representation of the WRKY transcription factors in the high biomass group showed the crucial roles in regulating genes accounting for assimilates accumulation and modulating the growth processes. Owing to their dual role, they can either mediate activation or act as repressors of target genes. For example, in rice, *OsWRKY72*, and *OsWRKY77*, they activate ABA signaling and repress GA signaling. Qiao and co-workers reported the direct role of *WRKY* in ABA signaling and drought-mediated regulation responses. Seven out of 45 *WRKY* TFs were downregulated in high-biomass groups, whereas 38 TFs were upregulated in high biomass [28]. All 16 *TIFY* were upregulated in the HB group, whereas 21 *NAC* TFs were downregulated among 38 differentially expressed TFs. Among 52 *bHLH* differentially expressed TFs, 6 were downregulated while 46 were upregulated in the HB group. *bHLH* TFs have a binding site for regulating MeJA inducible genes and are also reported to be transcribed in root apical meristem as a target of ARF5 downstream in the auxin signaling pathway [29]. Auxin-responsive TFs containing the B3 domain were also differentially expressed, and 12 out of 18 *ARF*s were upregulated in the HB group. Moreover, most members of *HSF* (Heat Shock Factors) were downregulated in the HB group, which shows the inactivated immunity and growth inhibitory processes responses.

### 2.10. Expression Profiles of Transcription Factors and Protein Kinases (Kinome)

Similarly, protein sequences of DEGs were subjected to the identification of protein kinases (PK) from an online database, iTAK. In *Sorghum bicolor* and *S. spontaneum*, kinome comprised 1210 PKs for Sbi and 2919 PKs besides allelic copies and segmental duplications [30]. From 8059 genes, 132 genes encoding for PKs were identified from an online database, iTAK. After reconfirmation by BLAST similarities, a heatmap of all the PKs having |log2FC>2|, showing 40 (30%) PKs as downregulated and 70% were upregulated was generated (Figure 5B, Supplementary file 2; Table S9). Among 132 PKs, 104 (78%) were receptor-like Kinase (*RLK*s), 6 PKs (4.8%) were calcium- and calmodulin-regulated kinase (*CAMK*) 7 (5.3%) sequences coding cyclin-dependent kinase-like kinase (*CMGC*) and other families belonged to serine/threonine kinase (*STE*), tyrosine kinase-like kinase (*TKL*) etc. RLKs are the kinases having a P Kinase domain, either membrane localized (*RLK*) or membrane-associated proteins localized in cytoplasm close to the plasma membrane (*RLCK*). Several Pelle (*RLK*) kinase proteins are implicated in cell wall metabolism, hormone signaling, and growth-related responses. Similarly, *CAMK* and *CGMC* protein kinases are involved in the cell cycle and certain growth responses.

### 2.11. Hormone Quantification

**Figure 6:**
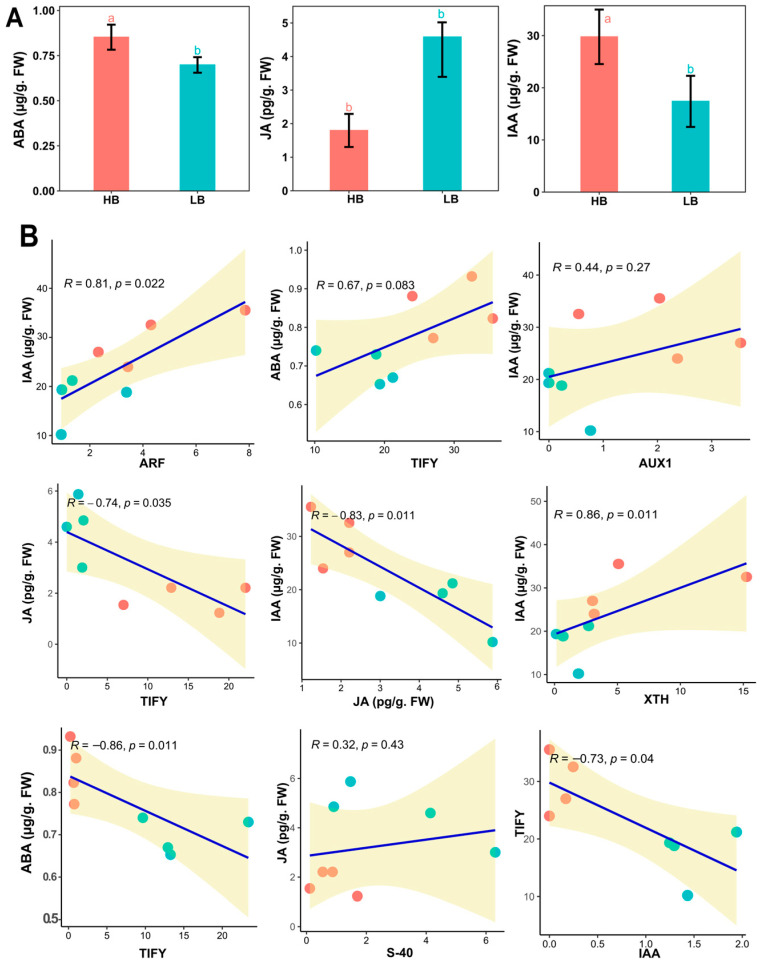
(**A**) Concentrations of the endogenous hormones, i.e., ABA, JA, and IAA, in the leaves of high and low biomass F2 segregants. Bar charts present the means with error bars showing standard errors, different letters are based on one-way ANOVA and LSD tests at α = 0.05. (**B**) Linear regression model of hormone content and qPCR values of the genes identified in RNA-Seq. analysis in the respective signaling pathway.

To confirm the empirical evidence of FPKM profiling in the signaling of IAA, ABA, and JA of the hormones, we quantified three hormones from the plant leaf samples (Figure 6A). IAA and ABA concentrations were high (41.32%, 18.82) in high biomass samples. Meanwhile, JA contents were drastically high in low biomass samples by 155.86%. This shows a highly activated defense system in the low biomass group, which may hinder the activity of growth-responsive genes. Further, linear regression between hormone concentration and hormone signaling genes was performed, which showed auxin concentration has a positive linear relation with *AUX1*, *ARF*, and *XTH*, whereas a negative relationship was observed in JA (Figure 6B).

### 2.12. RNA-Seq Data Validation by qRT-PCR

**Figure 7:**
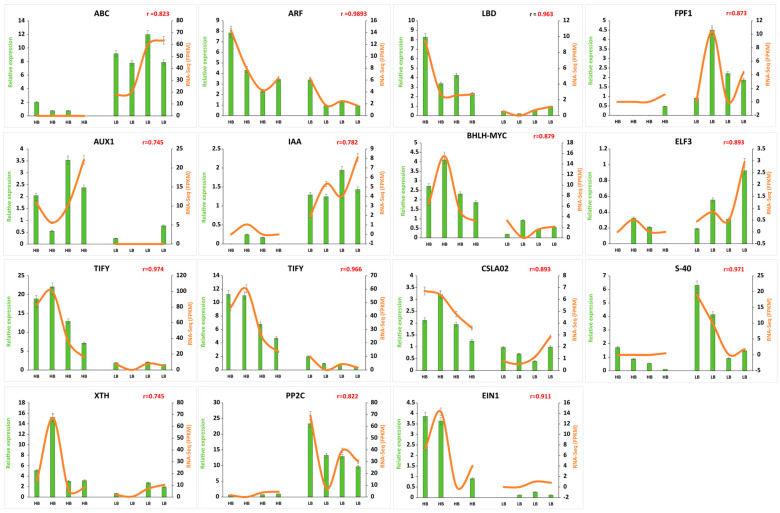
Confirmation of FPKM by qPCR expression. Green bars represent the relative expression in qPCR, and orange lines represent FPKM values in transcriptome for corresponding genes. Values on the *y*-axis indicate relative expression levels of qPCR (**left**) and RNA-Seq (**right**). Error bars show standard error of the means at (*p* < 0.05), and “r” is indicative of correlation between qPCR and FPKM expression values.

To validate expression patterns of genes identified by RNA-seq data, 15 genes were selected and examined by qRT-PCR (Figure 7). One gene related to *ABC* transporter (Soffic.10G0011210-1A), four genes from auxin signal transduction pathway (Soffic.10G0011210-1A, Soffic.03G0032240-1A, Soffic.10G0012000-1A, Soffic.01G0033270-1A), two JA (*TIFY*), 1 ABA (*EIN*) confirmed differences in transcript abundances in high and low biomass group. Similarly, cell wall synthesis and remodeling genes (*CSLA2*, *XTH*) were highly expressed in the high biomass group. Moreover, flowering and senescence-related genes, i.e., *ELF3*, *FPF1*, and *S-40* (Soffic.03G0016710-4D, Soffic.03G0036590-2B, Soffic.03G0024310-3H) showed consistent expression patterns in RNA-seq and qPCR quantification.

## 3. Discussion

Sugarcane holds a central position as first and second-generation feedstock crops in a bio-based energy generation system. Therefore, sugars, as well as biomass accumulating traits/factors, are of equal importance in bio-energy-based industries. Previously, sugarcane genomics research was hampered owing to its polyploid and heterozygous nature, but recently published information-rich allele-specific sugarcane genome opened a range of possibilities for advances in molecular and genetic studies [31]. In our previous study, we made crosses between two species *S. officinarum* and *S. robustum*, and several rounds of field investigation showed that the integration of traits from both *S. officinarum* and *S. robustum* led to extreme biomass yield segregants (Supplementary file 1; Table S1). The biomass yield of domesticated and cultivated species *S. officinarum* is substantially higher than that of wild species *S. robustum*, but the high biomass yield F2 extreme segregant exhibited more than twice the biomass yield of cultivated “LA Purple”. The genomic basis of such high biomass yield could be the combination of favorable multiple alleles from these autooctoploid genomes of both cultivated and wild species, in addition to possible heterosis.

To dissect allele-specific genetic controls for extreme biomass yield, we analyzed the transcriptome profile of the F2 population using an allele-defined *S. officinarum* genome. Several differentially expressed GMs, alleles, and duplicated genes (tandem and paralogs) related to signal transduction, carbon metabolism, and protein synthesis were linked to modulations in biomass accumulations. Regarding hormone signaling pathways, we identified that pivotal regulatory members of the signaling cascade, i.e., repressors of auxin, jasmonic acid, and abscisic acid, performed a principal role in hormone signaling dynamics in the high biomass group (Figure 4A). Moreover, results depicted the influence of auxin regulation on certain carbohydrate-related metabolism genes and predicted the possible role of protein kinases and transcription factors in regulating growth and biomass accumulation processes (Figure 5).

Accumulation of plant biomass with gradual growth and maturity is a genetically regulated process involving complex crosstalk of hormones. Based upon KEGG enrichment analysis, several GMs, alleles, and duplicated genes belonging to auxin families were overrepresented in the high biomass group, indicating auxin-mediated growth for the accumulation of biomass. In the presence of auxin hormone, the transcript level of three gene families—AUXIN/IAA (Aux/IAA), SMALL AUXIN-UP RNAs (SAURs), and GH3—were transiently induced [32]. Additionally, the 26-S-mediated ubiquitination process led to the breakdown of Aux/IAA proteins, which is evident by the downregulation of two Aux/IAA (Soffic.10G0012000-1A (GM), Soffic.10G0013930-3C (allele)) members. Upregulation of SAURs is typically associated with accelerated cell elongation at juvenile and during different stages of floral organ development [33,34,35], which is evident in juveniles. On the other hand, increased SAUR expression is also an indication of inhibited activity of *PP2C*, which ceases the phosphorylation of H^+^-ATPases leading to decrease in apoplastic pH, thereby activating expansins, xyloglucans, pectinesterase and other cell wall-modifying proteins (Figure 3A,B). The activation of H^+^-ATPase activity also favors the hyperpolarization of the plasma membrane, thus boosting solute and solvent uptake turgor pressure, leading to cell expansion [36]. Similarly, the *GH3* family of the auxin signaling pathway encodes acyl—amido synthetases, which regulate the homeostasis of auxin by uniting auxin with free amino acids or mediating catalysis of conjugation [37]. Downregulation of *PP2C* and upregulation of *SAUR* support the hypothesis that SAURs repress the transcription of *PP2C* by creating the acidic PH of apoplast, which favors the cell elongation and expansion processes (Figure 7). The core findings from the gene expression and functional profiles concluded the interplay of auxin repressor (*Aux/IAA*) and abscisic acid repressor (*PP2C*), yielding distinct biomass phenotypes. To further investigate the auxin biosynthesis pathways and their regulation, we searched various plant databases related to plant hormones. The Arabidopsis Hormone Database (AHD) (https://ngdc.cncb.ac.cn/ (accessed on 11 July 2024)), Hormone database, (http://pgl.gnu.ac.kr/hormoneDB/ (accessed on 11 July 2024)), Hormonometer, (https://doi.org/10.1104/pp.109.138289 (accessed on 11 July 2024)) and the Plant Hormone Database (PHGD), provided comprehensive information on hormone-related genes, including those involved in auxin biosynthesis, transport, and signaling. These databases compile extensive data on the functions and interactions of hormone-related genes, facilitating a deeper understanding of their roles in plant development and stress responses. Unfortunately, the Auxin Database, which was specifically built two decades ago for computer models connecting auxin carrier distributions along with auxin concentrations and leading to auxin signaling, is no longer functional (https://hope.simons-rock.edu/~ekramer/AuxPara/welcome.html (accessed on 11 July 2024)). Therefore, by integrating data from the Plant Hormone Database (PHGD) with our RNA-Seq analysis, we identified key genes and regulatory mechanisms involved in auxin-mediated growth and biomass accumulation in sugarcane (Supplementary file 1; Figure S7). The study highlights the key roles of Tryptophan Aminotransferase of Arabidopsis (*TAA*) and *YUCCA* (flavin-containing monooxygenase) families in the auxin biosynthesis pathway. *TAA1* catalyzes the conversion of tryptophan to indole-3-pyruvic acid (*IPA*), which *YUCCA* enzymes then convert to indole-3-acetic acid (IAA). This two-step pathway is well-documented in *Arabidopsis* and conserved across various plant species, including sugarcane [38]. Based on classical knowledge, auxin produced in meristematic cells primarily influences the transition to flowering, mainly by the action of LBD genes. The lower expression of LBD genes on most of loci might be an adaptation to delayed flowering and prolong the vegetative growth phase, thereby contributing to higher biomass accumulation (Figure 3A). This finding aligns with the concept that auxin’s role in post-mitotic cells is to support sustained growth, whereas its role in meristematic cells is to trigger developmental transitions such as flowering [39].

Regarding JA, the downregulation of JA receptor protein *COI1* and upregulation of repressor genes (*JAZ*) indicated the arrested transcription of jasmonic acid-responsive genes. *JAZ* repressors containing the JASMONATE ZIM domain (*JAZ*) bind to specific transcription factors of JA by its conserved C-terminal domain, which also recruits *TOPLESS* co-repressors or the Novel INteractor OF JAZ (*NINJA*) [40]. In our data, 13 repressor alleles (TIFY) in the JA signaling pathway showed upregulation in high biomass, pointing toward hampered activity of downstream JA-responsive genes. JA is a critically important hormone in defense-related responses by synthesizing low molecular weight compounds, such as polyphenols, alkaloids, quinones, terpenoids, and polypeptides [41]. Conversely, the downregulation of JAZ repressors in low biomass indicates the active stress and immune response. Likewise, the downregulation of Coronatine-insensitive protein (*COI1*) indicated a lack of substrate for signaling, while upregulated JAZ repressor proteins inhibit stress-responsive gene activation. This repression, mediated by TOPLESS co-repressor, allowed normal growth-related gene function, promoting increased biomass in high biomass segregents. These results are complemented in another study that JA (150–250 µM MeJ) treated as elicitor causes the complete inhibition of biomass in medicinal plant *Withania somnifera* (L.) reported by [42], in *H. hirsutum* and *H. maculatum* [43] and *Centella asiatica* [44]. JA is involved in the inhibition of leaf expansion by repressing the activity of mitotic cyclin *CycB1*; 2 and cell division. JA signaling cascade, i.e., *COI1*-*JAZ*-*MYC2* in *Arabidopsis*, hampers the expansion of leaves [45]. The upregulation of GH3 genes in the auxin signaling pathway aided to deactivate JA by conjugation with amino acids like Asp, Met and Trp, promoting formation of adventitious roots [46]. Both jasmonate and auxin signaling processes depend on small protein ubiquitin and 26S proteasome complex (Figure 3 A, B).

Cell wall loosening and the addition of new monomer units are essential for growth, which is predominantly activated by the auxin hormone [47]. It is achieved through the activation of certain genes, i.e., *EXP*, *XTH*, and *ENDO-(1,4)*-β-D-*GLUCANASE*s (*CELLULASE*s). The upregulation of genes related to EXP (expensins) and CESA (cellulose synthase) indicates enhanced metabolism of the cell wall. Moreover, XTHs control the expansion of cell walls by hydrolysis and re-ligating xyloglucan polymers which are cross-linked with cellulose microfibers [48,49]. Significantly upregulated expression in XTH (XET, XEH) genes might indicate increased beta-xylosidase activity, which is rate limiting in xylan hydrolysis and involved in the thickening of the secondary cell walls (Supplementary file 1; Figure S6).

The higher activity of growth-responsive and cell wall-related genes accompanied by delayed reproductive maturity leads to increased biomass. Downregulation of flowering-related genes, i.e., FLOWERING PROMOTING FACTOR 1 (*FPF1*), EARLY FLOWERING 3 (*ELF3*), and AGAMOUS-LIKE MADS-BOX PROTEIN (*AGL12*, *SVP*) indicates extended vegetative growth and late maturity, leading to higher biomass and sugars metabolism in HB group. The most important floral organ identity-related and regulatory genes are MADS-box genes, which encompass numerous sub-families and transcription factors. Recently, a study performed on diploid and polyploid species of brassicaceae revealed that the MADS-box gene can convert annual flowering plants into polycarpic perennial plants. Further, dosage effects indicated that when three genes are functional, the plant maintains a robust polycarpic perennial phenotype, indicating their collective role in flowering evolution [50]. ScFT3 in sugarcane can rescue late flowering in *Arabidopsis* mutants, unlike ScFT5. It showed high expression in leaves during floral induction in short day-induced sugarcane plants, suggesting a role in initiating flowering alongside other FT-like genes in sugarcane [51]. Inflorescence and senescence are the sister stages, with the latter as an important terminal stage. Senescence-related *NAC* transcription factors and a newly identified S-40 gene family also showed downregulation (Figure 3B and Figure 5A) [52,53]. The expression levels of flowering and senescence-related genes support the notion that the HB group undergoes an extended vegetative phase and delayed reproductive maturity. The shift of gene expression patterns, i.e., from vegetative to reproductive, showed the onset of early reproduction maturity in low biomass samples [54]. Similar results were reported by Habiba et al. in rice, where CRISPR/Cas9 knockout of OsS40 five mutants led to delayed senescence (stay-green phenotype), altered grain traits, differential regulation of senescence-related and SWEET genes, and reduced chlorophyll degradation [53]. Hormone pathways and cell signaling processes also recruit multiple TFs, protein kinases, and phosphatases for the regulation of growth and development. Among many TFs differentially regulated in our study, *ARF*, *bHLH*, *MYB*, *WOX*, and *WRKY* are closely related to hormone signaling. Twelve out of eighteen transcription factors were upregulated in high biomass, which indicates that they activate participation in ARF-mediated transcription of target genes and is critically important for auxin signal transduction. ARF proteins can either be transcriptional activators or repressors of downstream genes, which is determined by the structure of the MR domain of corresponding genes [55]. In our data, 91% of bHLH TFs were upregulated in high biomass, which interact with *MYC* TFs in jasmonic acid signaling, and upregulation of bHLH indicates the repressed activity of JA signal transduction and responsive genes in high biomass group A study provided evidence that gain in function mutant ABA-INDUCIBLE BHLH-TYPE TRANSCRIPTION FACTOR/JA-ASSOCIATED MYC2-LIKE1 (*JAM1*) showed downregulation of JA responses, reduced root growth and anthocyanin accumulation. The *MYB* proteins showed mixed responses, indicating 40% upregulation in high biomass. MYB proteins are present in all eukaryotes and play an important role in primary and secondary metabolism, cell fate determination, and developmental processes.

Phytohormone metabolism also involves the activation of certain protein kinases, which are critical for the accumulation of sucrose and culm development processes [56]. Out of 132 protein kinase-related genes, *RLK* was highly regulated in high biomass, whereas *CAMK* and *CGMC*-related kinases were also upregulated in the high biomass group. Among *CAMK*, calcium-dependent protein kinases are very important in sugar metabolism and hormone signaling [57]. Sucrose synthase is an enzyme that undergoes the conversion of sucrose from glucose and fructose, whereas *CDPK*s phosphorylate sucrose synthase and regulate their biological activity [58]. CBL-interacting protein kinases (*CIPK*s) are also calcium sensors; due to their structural similarity, these proteins are regarded as a subgroup of SNF-like kinases, i.e., *SnRK2* and *SnRK3* [59]. Most of the members of *SnRK2* and *SnRK3* in our RNA-Seq data were highly expressed in high biomass samples. Although ABA is known as a growth inhibitor [60], paradoxically, ABA in non-stressed conditions acts as a growth promoter [61]. Calcineurin B-like (*CBL*) kinases also bind to calcium and mediate certain growth and stress-related responses by interacting with *ABA* [62]. The process of ABA-stimulated growth is positively influenced by kinases and negatively induced by phosphatases (*PP2C*). ABA signaling also recruits calmodulin sensor protein to sense Ca^+^ for the regulated signaling and ABA-mediated enhanced growth. Upregulation of multiple *MAPK* kinases belonging to the *CGMC* category could be involved in sucrose metabolism as studies suggest that *ScMAPK-4* might be an important player in optimizing source–sink relations in sugarcane [63]. The upregulation of protein kinases like MAPK and CDPK in high biomass group suggests enhanced signal transduction processes that promote growth. MAPK pathways are well-known for their role in stress responses and growth regulation, while CDPKs are involved in calcium-mediated signaling crucial for various cellular processes. The downregulation of LRR family kinases in high biomass samples could point to a reduced capacity for pathogen resistance mechanisms, which are often mediated by these receptors, in favor of growth-promoting pathways. Hormone metabolism is regulated by multiple kinases, transcriptional factors, and regulators (Figure 5A,B and Figure 6A), and the crosstalk between the hormones regulates growth and development-related responses. In summary, the qPCR analysis reveals that HB samples consistently exhibit higher gene expression levels among various genes compared to LB samples. The correlations between growth-responsive gene expression and biomass are strong, indicating a robust relationship between these variables. Statistically, the correlation values ranged from 0.745 to 0.9893, underscoring the significant impact of gene expression levels on biomass. Moreover, the expression profiles in Allo or autopolyploid hybrids are the mosaic of the gene variants, and these results can be used to achieve preferential phenotype using bioengineering techniques for candidate gene variants (Figure 7).

## 4. Materials and Methods

### 4.1. Background of RNA-Seq Reads

*S. officinarum* was crossed with *S. robustum* species, which is indigenous to New Guinea. Two genotypes, “male sterile LA Purple” (2n = 8× = 80) and “MOL5829” (2n = 8× = 80), were selected as representatives of the two species, respectively. Among 98 F1 progenies, 20 plants of F1 progeny were selected and self-fertilized after the measurements of stem weight, diameter, and sugar contents. Later on, 272 F2 progenies were field-tested at different stations, and 120 plants with extreme biomass were chosen for further experimentation. Of the 98 F1 progenies, 20 were selected and self-fertilized after assessing stem weight, diameter, and sugar content. Subsequently, 272 F2 progenies were field-tested, and 120 plants with extreme biomass were chosen for further study. Field trials with three biological replicates were conducted at the Hawaii Agriculture Research Center Kunia station, Oahu, Hawaii. Among the 120 F2 plants, 22 progenies (14 high biomass and 8 low biomass) were selected for RNA-seq analysis based on their three-year average biomass measurements. (Supplementary file 1; Table S1 and Figure S1). These 22 F2 progenies were classified as high and low biomass groups based on their three-year average biomass measurements: those F2 plants having 75th or above percentile of a three-year average dry weight were grouped as high biomass group (14 F2 progenies), and F2 plants underneath the 25th percentile were classed in the low biomass group (8 F2 progenies). Finally, we selected 22 plants with extremely high and low biomasses for transcriptome analysis. The first dewlap leaf, stem internodes from 3rd, 9th, and 15th node (internode at the first dew lap leaf was numbered as first internode) were collected from 8-month-old F2 progenies. Plant tissues were ground to powdered form in liquid nitrogen, and 100 mg sample was used for RNA extraction using Omega Bio-tek E.Z.N.A.^®^ Plant RNA kit (Omega Bio-tek, #R6827–02, Norcross, GA, USA). Finally, samples from internodes and leaves were pooled from individual plants, and 1ng of RNA was used for Illumina RNA sequencing. All plant materials used for RNA-seq studies were obtained from the Hawaii Agriculture Research Center Kunia substation (Waipahu, HI, USA) and Texas A&M AgriLife Research and Extension Center at Weslaco (Weslaco, TX, USA). The nutrient supplication in the case of nitrogen was 112–134 kg/ha (approximately 100–120 lbs/acre), applied in three splits as basel and subsequent two splits. Phosphorus and Potassium were administered at a rate of 45–67 kg/ha and 134–179 kg/ha following the recommendations from Louisiana Farm Bureau News. Moreover, Sulfur was applied at 22–34 kg/ha in accordance with the guidelines from ICL Growing Solutions (https://icl-growingsolutions.com). The field sites in Hawaii and Texas provided varying day lengths, which are typical of tropical and subtropical regions. The plants experienced natural daylight cycles, with day lengths ranging from 10 to 14 h depending on the season. Light intensity was averaging around 1000–1200 µmol m^−2^ s^−1^ during peak sunlight hours. The detailed methodology of the field experiment and physiological attributes are described in the article [22].

**a.**  **Sequence read alignment and analysis of differentially expressed genes**

Paired-end raw RNA-Seq reads sequenced by HiSeq2500 were used for analysis, with NCBI BioProject ID PRJNA347369 and Short Read Archive under SRR5223340–SRR5223361. FastQC was used for the initial visualization to access the initial quality of reads, i.e., GC content, Per Base Sequence Quality, and adapter Sequence (http://www.bioinformatics.babraham.ac.uk/projects/fastqc (accessed on 2 January 2022)). We used trimmomatic to remove the low-quality reads [64]. Trimmed reads were aligned to *S. officinarum* genome LA Purple’ (2n = 8× = 80) using STAR (V. 2.7.11a) [65] (https://github.com/alexdobin/STAR) and HISAT2 (V. 2.2.0) (http://ccb.jhu.edu/software/hisat2 (accessed on 13 February 2022)) [66]. Following HISAT2, stringtie and DeSeq2 pipeline were used for analysis, whereas, additionally, STAR was used as an aligner for indexing and alignment of RNA-Seq paired-end reads. StringTie was used to assemble STAR-aligned transcripts to reference genome followed by quantification of transcripts (http://ccb.jhu.edu/software/stringtie or https://github.com/gpertea/stringtie (accessed on 15 February 2022)) [67]. Differential expression analysis (DEGs) was performed using DESeq2 (V. 1.16.1) [68], a bio conductor package in R software. Differentially expressed genes were sorted out using the criteria of FDR value| ≤0.05|, *p*-value |<0.01| and |log2FC >2, < −2| for up and downregulated DEGs. Partial least squares-discriminant analysis (PLS-DA), hierarchical clustering, and volcano plot were processed from FPKM and fold change and visualized using R-statistical package (V. 3.6).

### 4.2. Functional Annotation and GO Enrichment Analysis

Gene ontology (GO) enrichment analysis was conducted using OmicShare (https://www.omicshare.com (accessed on 16 May 2024)) and TBtools (https://github.com/CJ-Chen/TBtools (accessed on 16 February 2022)). GO terms with adjusted *p*-values less than 5% were considered significant and visualized using REVIGO [69] (http://revigo.irb.hr/ (accessed on 16 May 2024)).

### 4.3. KEGG Analysis, Transcription Factor and Kinome

Dynamic KEGG enrichment analysis (Kyoto Encyclopedia of Genes and Genomes) of genes was performed using “omicsahre” to identify functional annotations and signaling pathways [70] (https://www.omicshare.com (accessed on 16 May 2024)) which were later confirmed with blastn in Phytozome (https://phytozome-next.jgi.doe.gov/ (accessed on 2 March 2022)). From the results of KEGG, hormone, carbohydrate metabolism, and other genes related Furthermore, transcription factors (TF) and protein kinases (PKs) within the genes were identified using ITAK, an online database (http://itak.feilab.net/cgi-bin/itak/index.cgi (accessed on 8 April 2022)) and visualized by TBtools [71,72].

### 4.4. Weighted Gene Co-Expression Analysis (WGCNA) Analysis

WGCNA was conducted in R to identify co-expression patterns among DEGs. Hierarchical clustering was performed using Ward’s method [73], and unsigned topological overlap matrices (TOM) were created. We created adjacency matrices using the adjacency function and soft power threshold as eight and with a merge cut height as 0.25 and 0.30, in Dynamic Tree Cut (V. 1.63-1.), library in R, From the module–trait relationship heat map, highly correlated significant (r > 0.83, 0.91) modules “blue” and “greenyellow” were used to construct the networks in cytoscape (v. 3.9.1) [74].

### 4.5. Hormones Quantification

In total, 5 samples from each group were selected from high and low-biomass samples. The first dewlap sugarcane leaf was selected, and its middle region was used for the quantification of IAA, ABA, and JA, as previously stated by [75]. To quantify IAA, 50 μL of d5-IAA (indol-3-acetic-2,2-[^2^H_5_] acid), 50 μL of d6-ABA (2-cis, 4-trans-abscisic acid-[^2^H_6_] ABA), and 5 μL of H_2_-JA (dihydrojasmonic acid) were added in 50 mg of the freshly powdered samples priorly extracted with extraction solvent (2-propanol:H_2_O:HCl = 2:1:0.002) in a 2 mL falcon tube. The reaction mixture was shaken at 1 RCF (Relative centrifugal force) for 30 min at 4 °C. After this, addition of 1 mL dichloromethane and reshaking was performed for 30 min at 4 °C. The solutions were kept in micro-centrifuge at 4 °C followed by 19,722 RCF (Thermo Scientific, Microcentrifuge, Fresco 17 (75003424), Waltham, MA, USA) for 5 min centrifugation. After discarding the supernatant, 1.2 mL of solvent was concentrated using a nitrogen evaporator with nitrogen flow. The samples were dissolved in 0.1 mL methanol (70% *v*/*v*) and centrifuged at 19,722 RCF for 5 min. Finally, 50 μL of sample solution was injected into the reverse-phase for the quantification of acidic hormones by 6410 Triple Quad LCMS.

### 4.6. Quantitative RT-PCR-Based Quantification for Gene Expression

RNA was extracted from leaves using Trizol [76], and cDNA was synthesized using PrimeScript™ RT Reagent Kit (Kusatsu, Shiga, Japan) ith gDNA Eraser. Quantitative RT-PCR was performed using TB Green™ Premix Ex Taq™ II kit (Kusatsu, Shiga, Japan) on a BioRad CFX-96 system. The reaction volume was 20 μL, with 1 μL of cDNA, 1 μM of forward and reverse primers, and 10 μL of TB Green™ PCR master mix (Kusatsu, Shiga, Japan). The thermal cycling conditions included 95 °C for 3 mins, followed by 40 cycles of 95 °C for 10 s and 50 °C for 30 s. There were three biological replicates, and normalization was performed by reference gene, i.e., GAPDH [77]. The normalized expression level was calculated as L = 2^−ΔCt^ and ΔCT = CT_target_ − CT_reference_. The primers used in the experiment were listed in (Supplementary file 1; Table S2).

## 5. Conclusions

This study provided a broad view of many coding GMs, alleles, and duplicated genes expressing in extreme biomass segregants of *Saccharum* species at the terminal growth stage before harvest maturity. Our analysis revealed differential expression in 8059 genes (gene models (GMs), tandemly duplicated genes, and paralogs) and alleles, with 5540 upregulated and 2519 downregulated genes in high biomass segregants. Several genes and alleles of the auxin super family, jasmonic acid, and abscisic acid pathway were differentially expressed in extreme biomass groups. Regulatory expression of the repressor genes of these hormones (Aux, JA, and ABA) controls the signaling cascade of downstream-responsive gene loci. Auxin was identified as the core hormone in the modulation of growth by upregulation of genes downstream of the cascade. Genes related to the abscisic acid (ABA) signaling pathway, including protein phosphatases (*PP2C*s) and *SnRK2* protein kinases, showed mixed regulation, but key ABA response genes were downregulated in the high biomass group. The remarkable ontogeny of the high biomass group might be shaped by the coordination of the internal balance of phytohormones, i.e., activated auxin and inactivated jasmonic acid and ABA pathways. Expression profiles and WGCNA-based networks provided compelling evidence of the close association between hormone-related genes and those involved in cell wall dynamics (such as those encoding for *CESA*, *XTH*, and *EXP*). Conversely, in the lower biomass group, an elevated expression of flowering-related genes (*FPFI*, *ELF3*, and *S-40*) was observed, suggesting an activation of stress and early maturity-related pathways. Moreover, our results highlighted a plethora of growth regulatory genes, protein kinases, and transcription factors that trigger modulated growth in high biomass. Further experimentation based on the function of these genes will elucidate the basis of heterosis in contrasting F2 segregants in sugarcane. The insights gained from this study can be directly applied to breeding programs aimed at improving both biomass and sugar yield, making sugarcane cultivation more economically viable and efficient.

## Data Availability

The transcriptome reads sequenced in this study are available at NCBI with BioProject ID PRJNA347369 and Short Read Archive under SRR5223340–SRR5223361.

## Supplementary Materials

The following supporting information can be downloaded at https://www.mdpi.com/article/10.3390/plants13162247/s1. Supplementary_File1: Table S1: Indicating the F2 progeny IDs, their respective NCBI IDs, abbreviation used in the manuscript and the respective biomass group. In Table S1, rows show the deleted samples as they were not consistent in the expression patterns of their biomass group. Figure S1: Sugarcane genotypes grown at Hawaii Agriculture Research Center Maui and Kunia substations. The dry weight (in metric tons per hectare per 12 months) of each F2 progeny was measured. The 1st and 2nd-year crops were newly planted while 3rd plants were ratoon crops. ^a^ Average yield from 2-year measurements. Figure S2: Dispersion (A) and MA (B) plot. Dispersion plot shows the variation calculation by Poisson distribution; blue dots show final genes, and red dots show which fit in the curve. MA plot representing transformed data M (log ratio) and A (mean average) of all the genes. Figure S3: A flowchart indicating the top GO terms associated with molecular functions involved in cell wall carbohydrate metabolism. Figure S4: Heatmap of abscisic acid and ethylene-related genes in HB genotypes. Figure S5: Sample clustering (A) scale independence (B) Dynamic cut tree (C). (A) Clustering of samples to detect outliers using Ward’s clustering method. (B) Left panel showing scale free-index versus soft threshold power, which was chosen 8 as the curve index flattens at 8. Right panel shows the means connectivity against soft threshold power. (C) shows the dynamic cluster dendrograms of the genes falling in the selected modules from WGCNA. Gene clustering was performed using dissimilarity measure (1-TOM), branches representing highly interconnected links of genes belonging to different modules (colors in figure). Figure S6: Hypothetical figure showing the dual mechanism of XTH proteins acting on xyloglucan chains and increasing the molecular weights, remodeling the cell wall structure. Figure S7: Distribution of different members of hormone-related genes in *S. spontaneum*, auxin includes 1501, abscisic acid 1501, and jasmonate-related 1041 genes. Table S2: List of primers of different genes identified by RNA-Seq used in RT-PCR. Supplementary_File2: Table S3: List of the differentially enriched genes along with their log2FC and FPKM values. Table S4: List of the hormone and growth-related genes along with their log2FC and FPKM values. Table S5: Information of the differentially expressed gene models and alleles involved in different pathways in sugarcane. Table S6: GO enrichment analysis of upregulated and downregulated DEGs in two biomass groups, values in parentheses () shows *q*-value (*p* adjusted) for each GO term. Table S7: KEGG enrichment analysis of DEGs and number of enriched genes out of total genes: values in parentheses () show *q*-value for each KO term. Table S8: List of genes annotated as transcription factors (TF). Table S9: List of genes annotated as protein kinases (PK).
