# Supplementary material for: Allele-Specific Hormone Dynamics in Highly Transgressive F2 Biomass Segregants in Sugarcane (Saccharum spp.)
Source: Plants (Basel). 2024 Aug 13;13(16):2247. doi: 10.3390/plants13162247 (PMC11358940; doi:10.3390/plants13162247)
Supplement: Supplementary file 1 [file plants-13-02247-s001.zip › Supplementary_File1.pdf]

Supplementary Data

Table S-1. Indicating the F2 progeny IDs, their respective NCBI IDs, abbreviation used in the manuscript and the respective biomass group. In this table 5 rows show the deleted samples, as they were not consistent in the expression patterns of their biomass group.

F2 ID	NCBI ID	Abbreviation	Group
9-9251	SRR5223340	Deleted	High biomass
9-9183	SRR5223344	HB1	High biomass
9-9178	SRR5223345	HB2	High biomass
9-9169	SRR5223346	HB3	High biomass
9-9148	SRR5223348	HB4	High biomass
9-9126	SRR5223349	Deleted	High biomass
9-9117	SRR5223350	Deleted	High biomass
9-9078	SRR5223352	Deleted	High biomass
9-9053	SRR5223356	HB5	High biomass
9-9019	SRR5223357	HB6	High biomass
9-9010	SRR5223358	HB7	High biomass
9-9009	SRR5223359	HB8	High biomass
9-9006	SRR5223360	HB9	High biomass
9-9001	SRR5223361	HB10	High biomass
9-9246	SRR5223341	LB1	Low biomass
9-9197	SRR5223342	LB2	Low biomass
9-9190	SRR5223343	LB3	Low biomass
9-9163	SRR5223347	LB4	Low biomass
9-9095	SRR5223351	Deleted	Low biomass
9-9074	SRR5223353	LB5	Low biomass
9-9069	SRR5223354	LB6	Low biomass
9-9065	SRR5223355	LB7	Low biomass

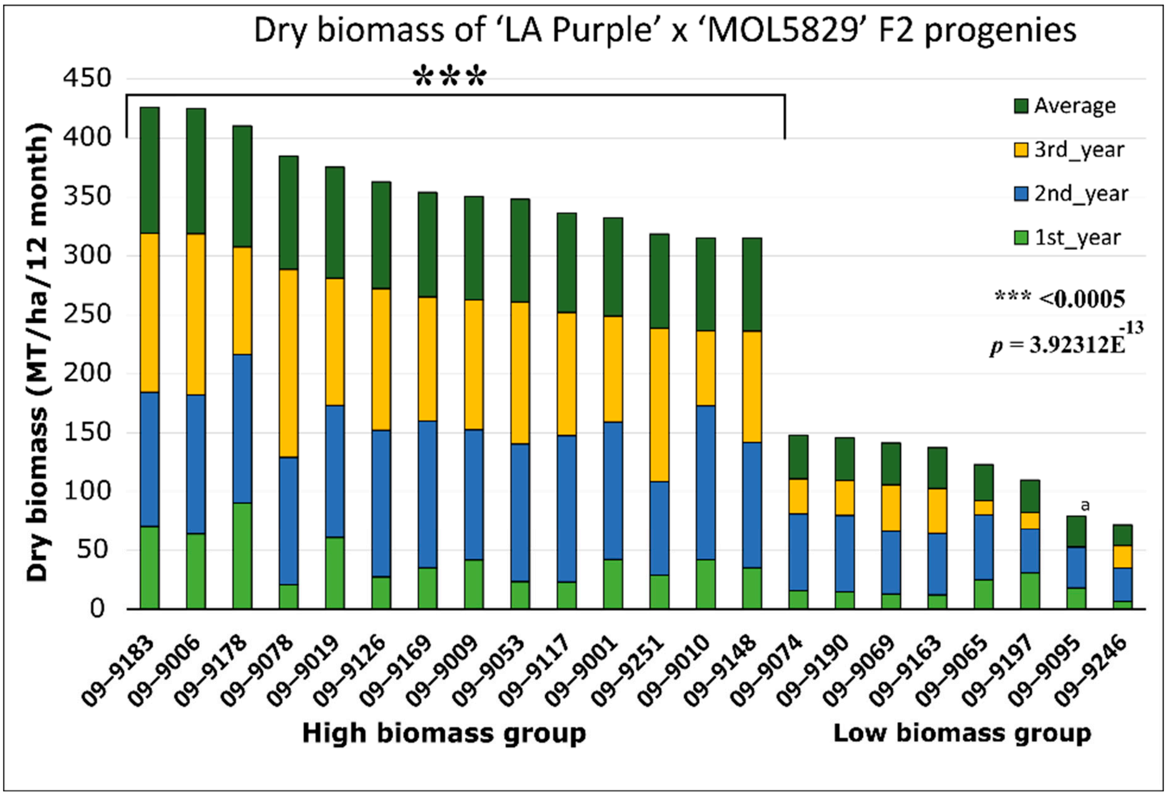

Figure S-1. Sugarcane genotypes grown at Hawaii Agriculture Research Center Maui and Kunia substations. The dry weight (in metric ton per hectare per 12-month) of each F2 progeny were measured. The 1st and 2nd year crops were newly planted while 3rd plants are ratoon crops.

[a] Average yield from 2 year measurements

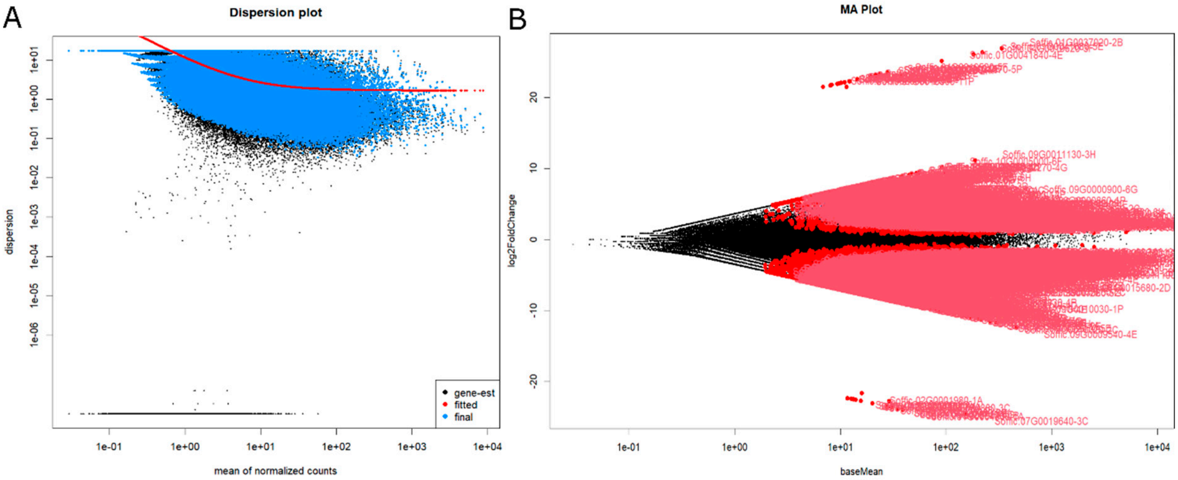

Figure S-2. Dispersion (A) and MA (B) plot. Dispersion plot shows the variation calculation by Poission distribution, blue dots shows final genes and red dots show which

fit in the curve. MA plot representing transformed data M (log ratio) and A (mean average) of all the genes.

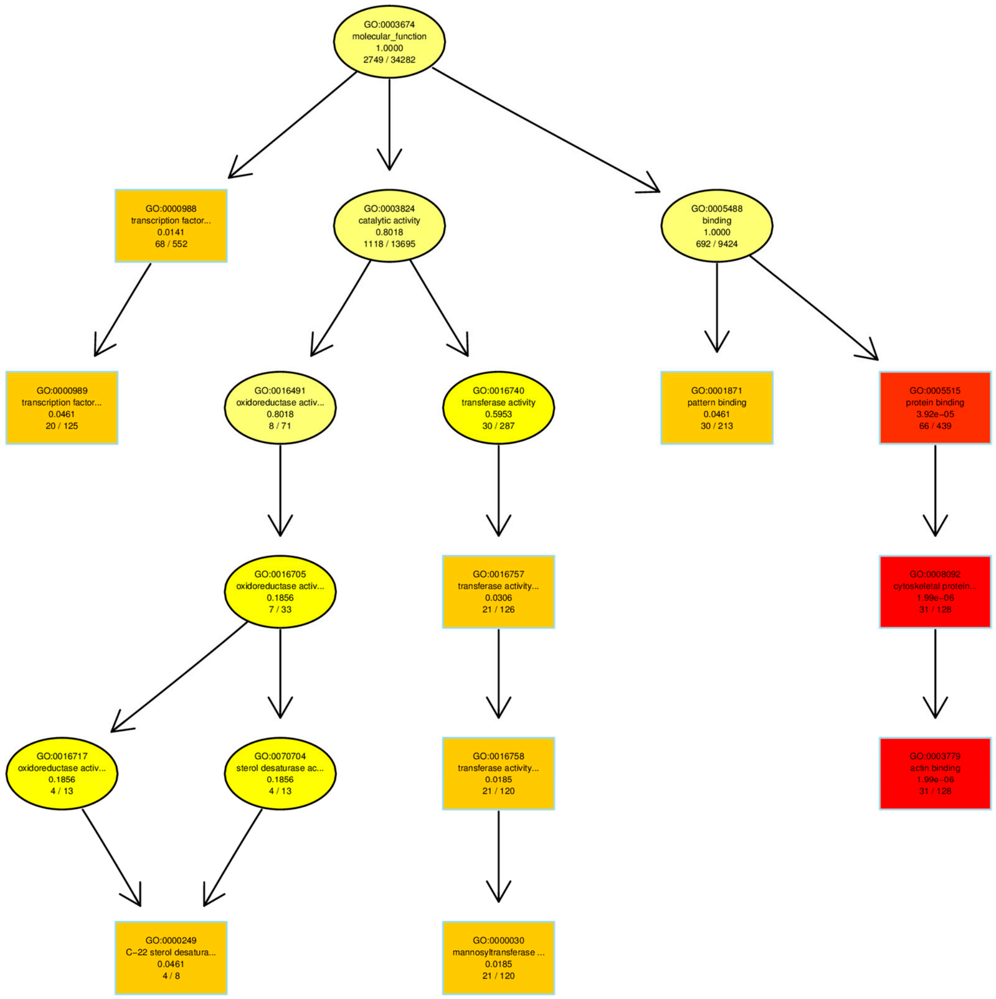

Figure S-3: Flowchart indicating the top GO terms associated to molecular functions involved in cell wall carbohydrate metabolism.

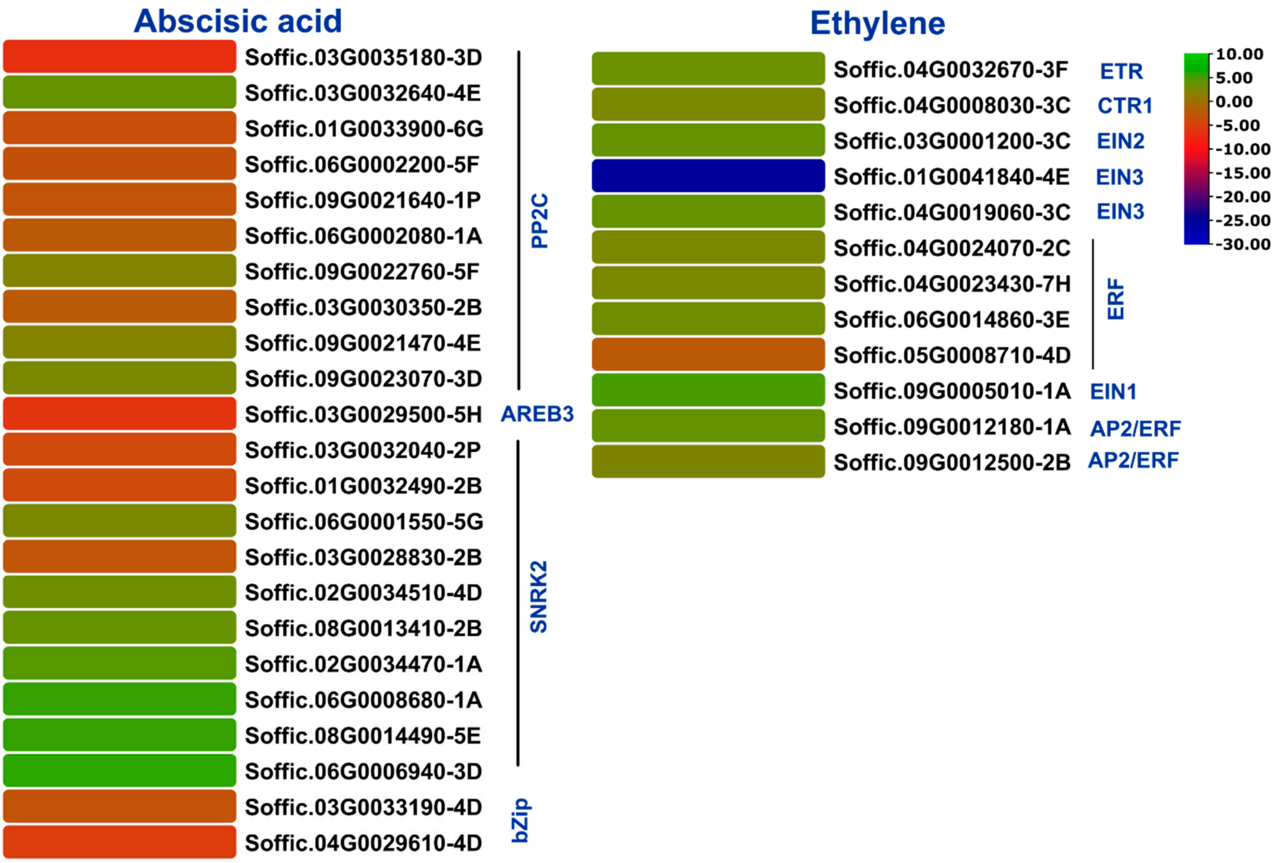

Figure S-4. Heatmap of abscisic acid and ethylene related genes in HB genotypes.

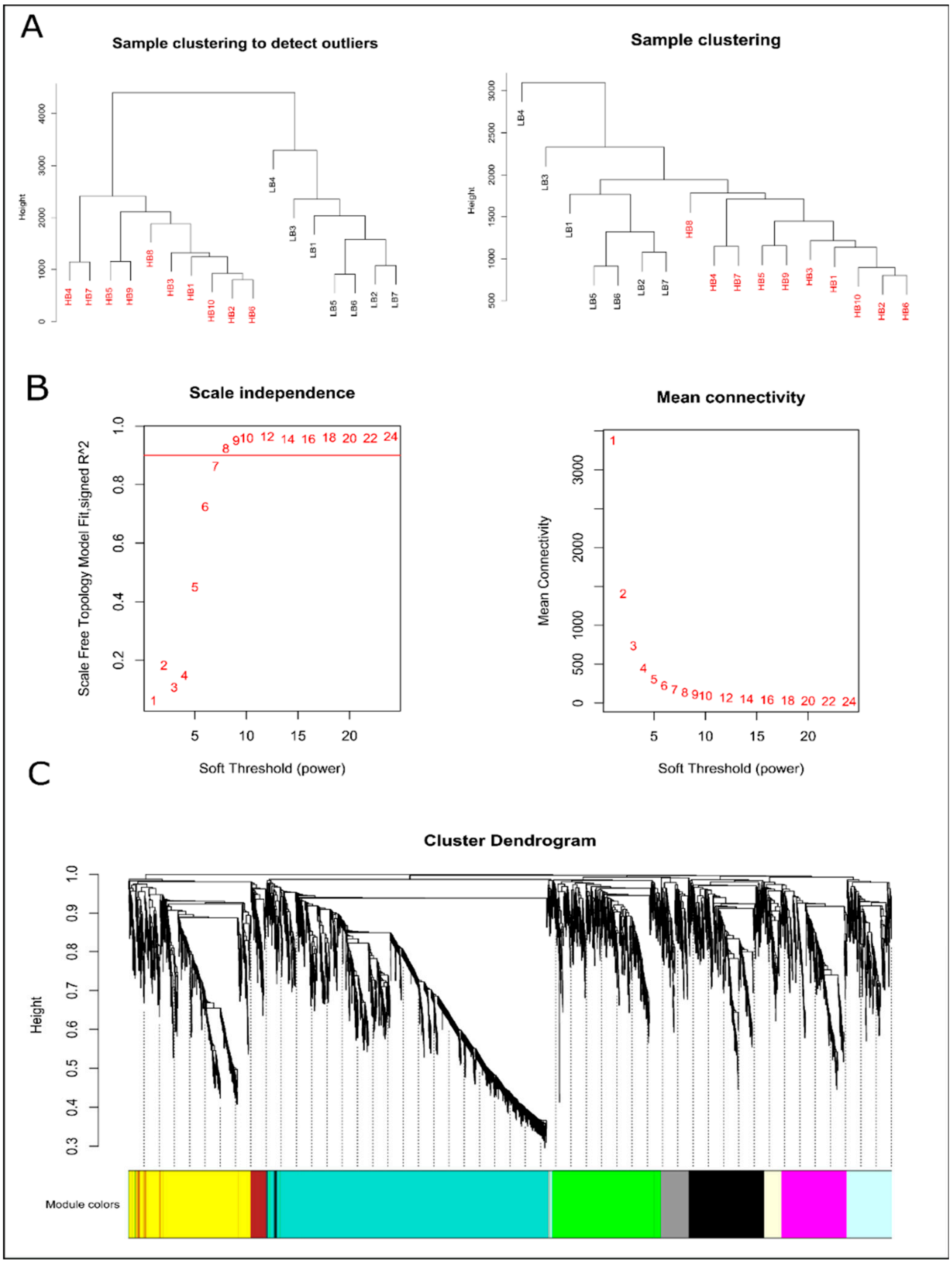

Figure S-5. Sample clustering (A) scale independence (B) Dynamic cut tree (C) A. Clustering of samples to detect outliers using wards clustering method B. Left panel showing scale free- index versus soft threshold power, which was chosen 8 as the curve index flattens at 8. Right panel shows the means connectivity against soft threshold power. C. shows the dynamic cluster dendrograms of the genes falling in the selected modules from WGCNA. Gene clustering was done using dissimilarity measure (1-TOM), branches representing highly interconnected links of genes belonging to different modules (colors in Fig.)

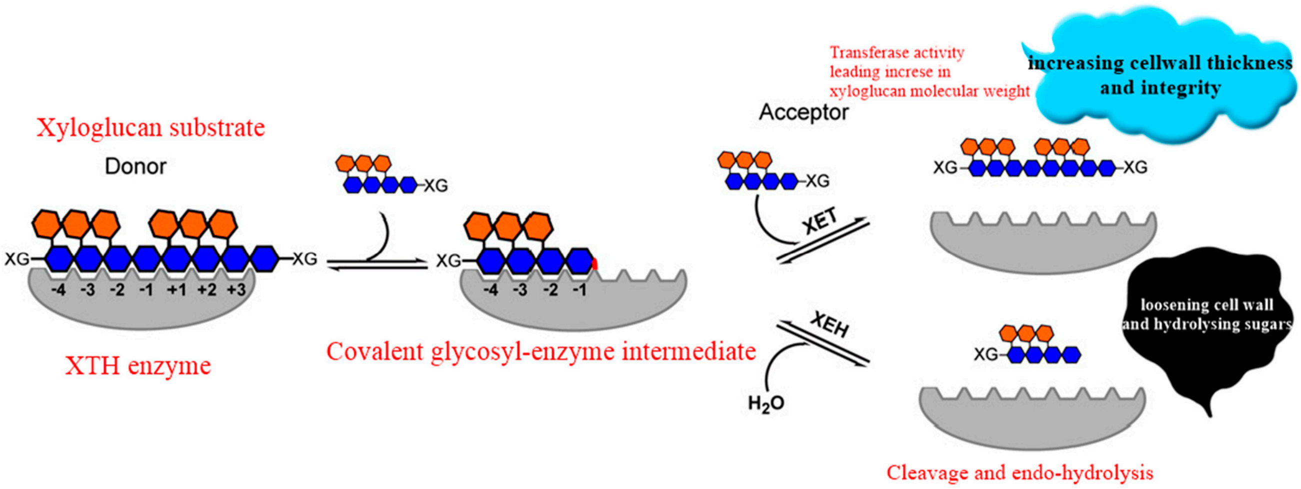

Figure S-6. Hypothetical figure showing the dual mechanism of XTH proteins acting on xyloglucan chains and increasing the molecular weights, remodeling the cell wall structure.

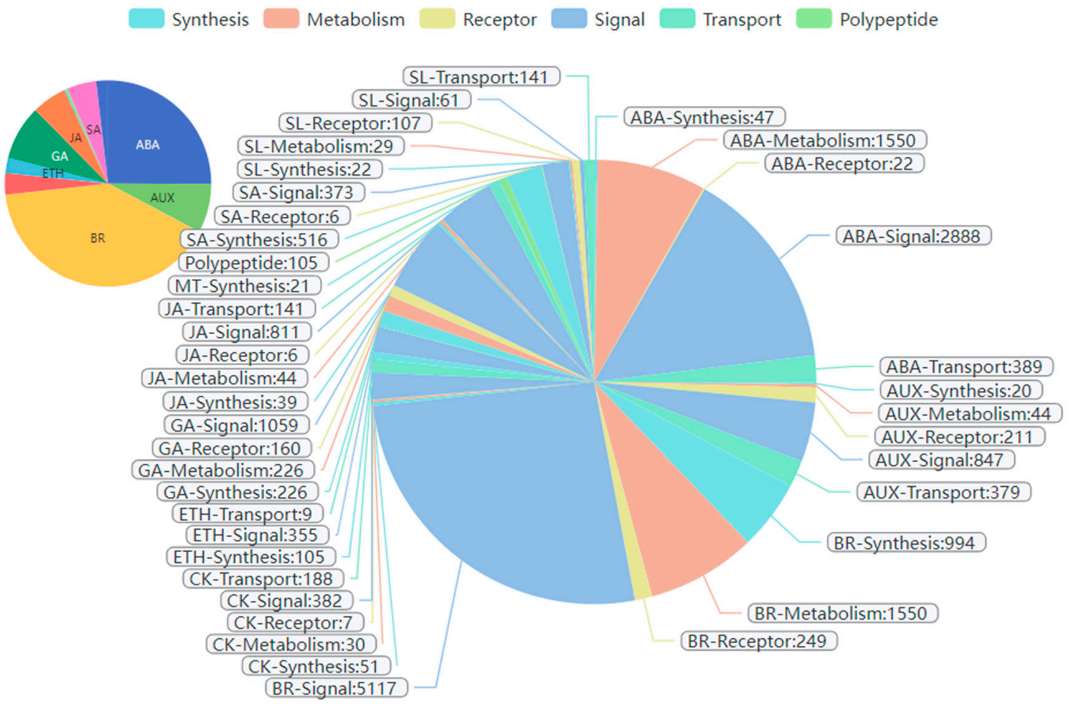

Figure S-7. Distribution of different members of hormones related genes in *S. Spontaneum,* Auxin includes 1501, Abscisic acid 1501 and jasmonate related 1041 genes.

Table S-2. List of primers of different genes identified by RNA-Seq used in RT-PCR

Gene name	Gene ID	Forward Primers	Reverse Primers
**ABC**	Soffic.10G0011210-1A	TGTGGCATGCAAAACCCATG	CATGGGTTTTGCATGCCACA
**ARFs**	Soffic.10G0011210-1A	TGTGGCATGCAAAACCCATG	CATGGGTTTTGCATGCCACA
**AUX1**	Soffic.03G0032240-1A	CACCGTCTACATCATCCCGG	CCGGGATGATGTAGACGGTG
**IAA**	Soffic.10G0012000-1A	CAGGAACGGGACTACATCGG	CCGATGTAGTCCCGTTCCTG
**LBD**	Soffic.01G0033270-1A	CAACATCTACGGGACCCCTG	CAGGGGTCCCGTAGATGTTG
**TIFY**	Soffic.01G0026780-1A	GCTGCGAAAGGATGATCTGC	GCAGATCATCCTTTCGCAGC
**TIFY**	Soffic.01G0026760-1A	AGGCTGGGTGGATTCAATGG	CCATTGAATCCACCCAGCCT
**bHLH-MYC**	Soffic.06G0002320-1A	GGCTCAATGCAGCACATAGC	GCTATGTGCTGCATTGAGCC
**XTH**	Soffic.07G0007320-1A	CATCAGGTCCTTCAAGCGGT	ACCGCTTGAAGGACCTGATG
**CSLA02**	Soffic.10G0023760-5G	TGCACAAGAAGGGATGGGAC	TTCGGGTAGAGGTCCCTGAG
**PP2C**	Soffic.01G0033900-6G	ACTTCTTCGGGGTGTTCGAC	GTCGAACACCCCGAAGAAGT
**EIN1**	Soffic.09G0005010-1A	AATGCGGACTCCTATGCGAG	CTCGCATAGGAGTCCGCATT
**ELF3**	Soffic.03G0016710-4D	AGCATATGGTCCCTCCTGGT	ACCAGGAGGGACCATATGCT
**FPF1**	Soffic.03G0036590-2B	CCGGGGAGGTGATCATCCTA	TAGGATGATCACCTCCCCGG
**S-40**	Soffic.03G0024310-3H	GATGACCGGCTTCATCGAGA	GGATCTCGATGGGATCCGTG
